# Gut microbiota in cancer: From molecular mechanisms to precision medicine applications

**DOI:** 10.1002/imt2.70017

**Published:** 2025-03-19

**Authors:** Weihua Xiao, Qiong Chen, Chunjiao Liu, Yueer Yu, Tianliang Liu, Yang Jin, Haifen Ma, Shifu Chen, Chao Jiang

**Affiliations:** ^1^ Department of Pathology, Beilun Branch of the First Affiliated Hospital, College of Medicine Zhejiang University Ningbo China; ^2^ MOE Key Laboratory of Biosystems Homeostasis & Protection, and Zhejiang Key Laboratory of Molecular Cancer Biology, Life Sciences Institute Zhejiang University Hangzhou China; ^3^ State Key Laboratory for Diagnosis and Treatment of Infectious Diseases, First Affiliated Hospital Zhejiang University School of Medicine Hangzhou China; ^4^ Department of Tea Research Institute Zhejiang University Hangzhou China; ^5^ HaploX Biotechnology Shenzhen China; ^6^ LifeX Institute, School of Medical Technology Gannan Medical University Ganzhou China; ^7^ Institute for Cancer Genetics and Informatics Oslo University Hospital Oslo Norway

## Abstract

The gut microbiota–cancer interaction functions through multi‐level biological mechanisms, forming the basis for both diagnostic and therapeutic applications. Current technical and biological challenges drive the field toward precision medicine approaches, aiming to integrate multi‐dimensional data for optimized, personalized cancer treatments.

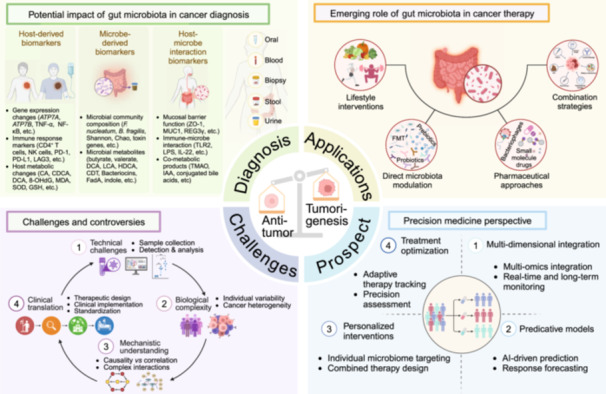

Cancer remains a major global health challenge. In recent years, the crucial role of gut microbiota in cancer development has gained increasing attention, with remarkable progress bringing new insights for cancer diagnosis and treatment. The gut microbiota participates in human metabolism, nutrient absorption, and various physiological processes while significantly influencing cancer initiation, progression, and therapeutic responses through its interactions with the host immune system. Given these diverse functions, understanding its underlying mechanisms has become essential for advancing cancer research and treatment.

## MECHANISMS OF GUT MICROBIOTA IN CANCER DEVELOPMENT

**Figure 1:**
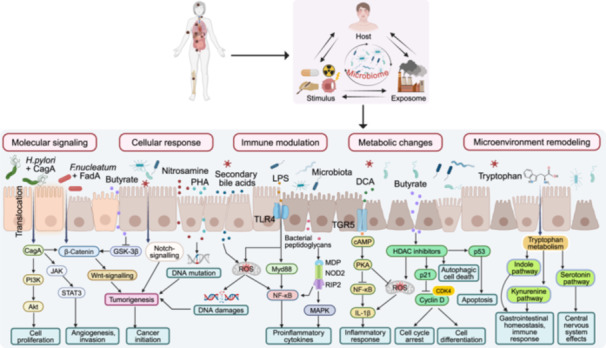
Gut microbes promote cancer progression by five core mechanisms. CA, cholic acid; CDCA, chenodeoxycholic acid; HDCA, hyodeoxycholic acid; MDP, muramyl dipeptide; MUC1, Mucin 1.

The gut microbiota, as the “second genome,” actively influences cancer development through diverse molecular and cellular pathways, making its role increasingly central to oncological research. Numerous factors like drugs, radiation, pathogens, and environmental exposures interact with the microbiota, influencing tumor formation. Research shows that gut microbiota contributes to cancer through molecular signaling, cellular response, immune regulation, metabolic changes, and microenvironment remodeling (Figure 1). This bidirectional interaction between the microbiota and host opens new opportunities for targeted interventions.

### Molecular signaling: Integration of bacterial‐induced signaling networks in cancer development

*Helicobacter pylori* cytotoxin‐associated gene A (CagA) activates multiple parallel pathways, including phosphatidylinositol 3‐kinase (PI3K)/protein kinase B (Akt) promoting cell proliferation and Janus kinase (JAK)/signal transducer and activator of transcription 3 (STAT3) driving angiogenesis and invasion [1]. *Fusobacterium nucleatum* (*F. nucleatum*) triggers β‐catenin and Wnt signaling through fusobacterium adhesin A (FadA) [2]. These pathways converge with glycogen synthase kinase 3 beta (GSK‐3β) regulation and interact with broader signaling networks, including Nuclear factor kappa B (NF‐κB), which can be activated through multiple routes, such as myeloid differentiation factor 88 (MyD88)—dependent pathways. The mitogen‐activated protein kinase (MAPK) cascade and IL‐1β signaling further contribute to this complex molecular network [3]. Together, these integrated signaling pathways orchestrate cell proliferation, angiogenesis, invasion, and cancer initiation.

### Cellular response: Direct microbial modulation of cellular phenotype and genetic stability

Microbial factors induce diverse cellular adaptations. *F. nucleatum* with FadA and butyrate modulate β‐catenin and GSK‐3β signaling, affecting cell behavior. Similarly, exposure to nitrosamines directly induces DNA mutations and damage, while secondary polycyclic aromatic hydrocarbons induce cellular stress responses through reactive oxygen species (ROS) generation [4]. These cellular responses culminate in sustained DNA damage and phenotypic transformations that drive tumorigenesis through multiple parallel pathways.

### Immune modulation: Bacterial activation of innate immune signaling and inflammatory responses

The immune response involves complex receptor‐mediated signaling cascades. Bacterial components, including lipopolysaccharide (LPS) and peptidoglycans, interact with toll‐like receptor 4, activating MyD88‐dependent pathways [5]. This activation subsequently engages nucleotide‐binding oligomerization domain‐2 and receptor‐interacting protein 2 signaling, leading to NF‐κB and MAPK pathway activation [6]. Beyond these interactions, the microbiota also regulates IL‐1β production through Takeda G protein‐coupled receptor 5, collectively orchestrating pro‐inflammatory cytokine production and inflammatory responses [7].

### Metabolic changes: Microbial metabolite‐mediated regulation of cellular fate and function

Microbial metabolites exert diverse effects through multiple pathways. Deoxycholic acid (DCA) engages cyclic adenosine monophosphate signaling to regulate ROS levels and modify cellular metabolism. Butyrate acts as a histone deacetylase inhibitor, influencing p21, cyclin‐dependent kinase 4 (CDK4), and Cyclin D pathways to control cell cycle progression [8]. These metabolic changes lead to various cellular outcomes, including cell cycle arrest, cell differentiation, and programmed cell death through both autophagic and apoptotic mechanisms [9].

### Microenvironment remodeling: Microbial influence on tissue homeostasis and systemic responses

The tissue microenvironment undergoes significant modifications through multiple metabolic pathways. Tryptophan metabolism branches into indole and kynurenine pathways, affecting gastrointestinal homeostasis and immune responses [10]. The serotonin pathway influences the central nervous system's function. These pathways collectively demonstrate how microbial activities comprehensively modify both the local tissue environment and systemic physiological responses, creating a complex network of host–microbe interactions.

## POTENTIAL IMPACT OF GUT MICROBIOTA IN CANCER DIAGNOSIS

**Figure 2:**
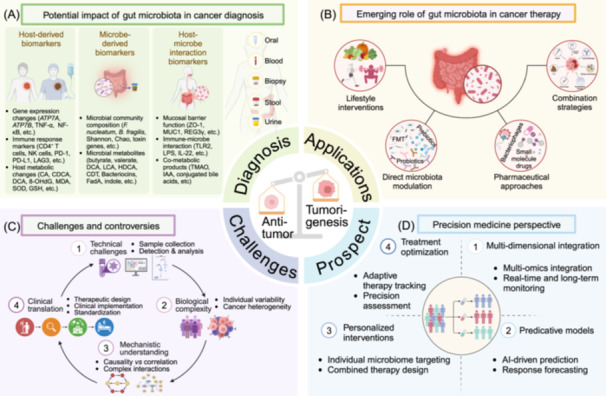
Applications, challenges, and precision medicine approaches of gut microbiota in cancer. (A) Host‐derived, microbe‐derived, and host–microbe interaction biomarkers for cancer diagnosis. (B) Therapeutic strategies for microbiota modulation in cancer treatment. (C) Current challenges in microbiota‐based cancer research. (D) Precision medicine perspective: from multi‐omics integration to personalized interventions.

Early cancer diagnosis is vital for effective treatment. The gut microbiota has gained recognition as a potential biomarker for cancer. Through intricate host–microbe interactions (Figure 2A), it can influence the tumor microenvironment, making it a promising avenue for early detection and diagnosis.

### Host‐derived biomarkers: Early indicators of cancer development

An imbalanced gut microbiota can alter gene expression, immune responses, and metabolic patterns, triggering early cancer signals. For instance, abnormal expression of copper transporters ATPase copper transporting alpha/beta (*ATP7A/B*) is linked to chemotherapy resistance in colorectal cancer (CRC; Figure 2A). *ATP7A* regulates copper homeostasis, and its disruption causes copper imbalance, leading to oxidative stress and cancer progression [11]. Although large‐scale studies of these gene markers are limited, small‐scale studies suggest differential expressions between cancer patients and healthy individuals. Emerging evidence suggests that copper homeostasis mechanisms could serve as novel diagnostic biomarkers, warranting further investigation into their clinical applications.

Chronic inflammation, characterized by the activation of tumor necrosis factor‐alpha (TNF‐α) and NF‐κB, contributes to the development of various cancers. Changes in immune response markers, such as reduced CD4^+^ T cells, impaired NK cell function, and immune checkpoint molecules like programmed cell death protein 1 (PD‐1), programmed death‐ligand 1 (PD‐L1), and lymphocyte activation gene 3 (LAG3), have been implicated in cancer progression [12]. These markers could serve as noninvasive indicators for early diagnosis (Figure 2A).

Host metabolic changes also provide valuable insights into early cancer detection. Elevated levels of microbial metabolites, such as DCA, and oxidative stress markers like 8‐hydroxy‐2'‐deoxyguanosine, malondialdehyde, and antioxidant indicators (e.g., superoxide dismutase and glutathione), are emerging as promising biomarkers for CRC screening and progression monitoring [13]. These metabolic alterations reflect changes in cellular homeostasis and DNA damage, making them useful for early diagnosis and potential therapeutic targets.

### Microbial‐derived biomarkers: Unveiling pathological mechanisms

Microbial biomarkers, comprising metabolites, proteins, and genes derived from microorganisms, provide valuable insights into cancer pathogenesis and progression. These molecules reflect both changes in microbial communities and specific pathogen‐host interactions, enabling early detection and disease monitoring.

Specific pathogens, such as *F. nucleatum* and *Bacteroides fragilis*, contribute to CRC progression through microbial‐derived factors like the FadA adhesin, which activates the β‐catenin signaling pathway. In addition, shifts in microbial community composition, including decreases in alpha diversity (e.g., Shannon and Chao indices), are associated with gastric cancer. The abundance of specific microbial genes, such as those encoding bile acid hydrolases, can help differentiate liver cancer patients from healthy individuals [14].

Microbial metabolites likewise play a significant role in cancer regulation [15, 16]. For example, butyrate suppresses tumor formation, but its dysregulation can lead to silencing tumor‐suppressor genes in CRC. Secondary bile acids like DCA and lithocholic acid (LCA) also influence cancer progression; LCA activates the farnesoid X receptor (FXR), promoting liver cancer cell invasion. Additional microbial metabolites, such as *F. nucleatum*'s cytolethal distending toxin, bacteriocins, and indole, show tissue‐specific distributions and offer diagnostic potential, further supporting their use as cancer biomarkers.

### Host–microbe interactions: Synergistic effects in the tumor microenvironment

Disruption of gut microbiota can compromise mucosal barrier integrity, thereby promoting cancer development through multiple mechanisms. The tight junction protein zonula occludens‐1, a critical component of the mucosal barrier, shows reduced expression in CRC, correlating with enhanced metastatic potential [17]. Additionally, decreased levels of the antimicrobial protein regenerating islet‐derived protein 3 gamma (REG3γ) facilitate microbial translocation across the barrier, resulting in chronic inflammation and subsequent cancer progression.

Abnormal immune‐microbe interactions play a significant role in tumor development. For instance, recognition of LPS by toll‐like receptor 2 (TLR2) receptors can trigger immune suppression by expanding myeloid‐derived suppressor cells and reducing antitumor immune responses. Additionally, the cytokine IL‐22 has a dual function in balancing epithelial repair and tumor growth, making it a potential therapeutic target for liver cancer.

Co‐metabolites, such as trimethylamine N‐oxide (TMAO) and indole‐3‐acetic acid, influence cancer progression through their impact on both tumor angiogenesis and immune modulation. Notably, TMAO, a product of choline metabolism, promotes angiogenesis in CRC but may suppress metastasis in breast cancer. Furthermore, microbiota‐regulated bile acid conjugation, particularly conjugated bile acids, affects the behavior of cancer stem cells and reshapes the immune microenvironment, supporting cancer progression and immune evasion [18].

In summary, the significance of gut microbiota in cancer extends beyond basic pathology, suggesting the need for ecological perspectives and synergy between microbiota interventions and current treatments.

## EMERGING ROLE OF GUT MICROBIOTA IN CANCER THERAPY

The gut microbiota offers multiple therapeutic strategies in cancer treatment through three main approaches (Figure 2B). First, lifestyle interventions, including a high‐fiber diet and moderate exercise, help maintain microbial diversity and enhance treatment efficacy. Second, direct microbiota modulation strategies encompass targeted antimicrobial therapy, probiotics, prebiotics, and fecal microbiota transplantation (FMT). Notably, FMT has emerged as a promising approach for restoring microbiota diversity in chemotherapy or immunotherapy patients, leading to improved therapeutic responses [19].

The third approach involves pharmaceutical interventions targeting microbiota‐host interactions. These include engineered oncolytic bacteria, phage therapy, and small molecule drugs that modulate microbial metabolism. Recent studies demonstrate that these interventions can significantly impact immunotherapy and chemotherapy outcomes, particularly through enhanced immune system activation and reduced treatment resistance [20]. The integration of these three approaches—lifestyle modification, direct microbiota modulation, and pharmaceutical intervention—represents a comprehensive strategy for improving cancer treatment outcomes.

## CHALLENGES AND CONTROVERSIES OF GUT MICROBIOTA IN CANCER DIAGNOSIS AND TREATMENT

Current challenges in microbiota‐cancer research span technical, biological, and clinical domains (Figure 2C). At the technical level, the complexity of microbial communities and their diverse metabolic outputs complicate the identification of cancer‐specific signatures. Biological challenges include environmental and genetic factors, along with underexplored fungal contributions that affect detection accuracy.

In therapeutic applications, FMT presents specific challenges regarding donor‐recipient compatibility and pathogen transmission risk in immunocompromised patients [20]. The uncertain stability of transplanted microbiota and limited long‐term follow‐up data pose additional obstacles to outcome prediction.

Moving from preclinical to clinical applications faces multiple barriers, including species‐specific variations, ethical considerations, and standardization requirements. Future progress depends on large‐scale longitudinal studies, advanced functional genomics focusing on the gut‐brain‐tumor axis, and standardized protocols. Integration of single‐cell genomic, single‐cell transcriptomic, and spatial transcriptomic technologies will be crucial for developing safer and more effective clinical applications.

## PROSPECTS OF GUT MICROBIOTA PRECISION MEDICINE RESEARCH

Recent technological breakthroughs and clinical advances show promise for incorporating gut microbiota markers into early cancer screening and personalized therapies, particularly for CRC (Figure 2D). Current research utilizes high‐throughput methods like 16S rRNA sequencing and metagenomics to map microbiota composition, though their resolution limitations affect causal inference. Advanced techniques, including single‐cell sequencing and metabolomics, are providing deeper insights into microbial–host interactions and metabolic networks.

The integration of microbiota research with precision medicine is advancing through multiple approaches: Artificial Intelligence (AI)‐driven predictive models, real‐time microbiome monitoring, and targeted therapeutic interventions. In vitro and in vivo functional studies of probiotics and fecal transplants are helping to elucidate specific microbial mechanisms, enabling more precise treatment strategies.

## CONCLUSION

The gut microbiota influences cancer development through multiple molecular and cellular mechanisms, presenting opportunities for both diagnostic and therapeutic applications. While challenges in standardization and biological complexity persist, emerging technologies and integrated approaches are advancing our understanding of microbiota‐host interactions. As research progresses, microbiota‐based strategies are likely to become valuable components of precision cancer medicine, contributing to improved patient outcomes.

## AUTHOR CONTRIBUTIONS

**Weihua Xiao**: Conceptualization; writing—original draft; investigation; methodology; funding acquisition; supervision. **Qiong Chen**: Conceptualization; methodology; writing—original draft; visualization. **Chunjiao Liu**: Data curation. **Yueer Yu**: Data curation. **Tianliang Liu**: Writing—review and editing. **Yang Jin**: Writing—review and editing. **Haifen Ma**: Writing—review and editing. **Shifu Chen**: Writing—review and editing; Supervision. **Chao Jiang**: Conceptualization; writing—review and editing; funding acquisition; supervision.

## CONFLICT OF INTEREST STATEMENT

The authors declare no conflicts of interest.

## ETHICAL STATEMENT

No animals or humans were involved in this study.

## Data Availability

Data sharing is not applicable to this article as no datasets were generated or analyzed during the current study. No new data and scripts were used for this commentary. Supplementary information (graphical abstract, slides, videos, Chinese translated version, and update materials) may be found in the online DOI or iMeta Science https://www.imeta.science/.
